# Fcγ receptor-mediated influx of S100A8/A9-producing neutrophils as inducer of bone erosion during antigen-induced arthritis

**DOI:** 10.1186/s13075-018-1584-1

**Published:** 2018-05-02

**Authors:** Irene Di Ceglie, Giuliana Ascone, Niels A. J. Cremers, Annet W. Sloetjes, Birgitte Walgreen, Thomas Vogl, Johannes Roth, J. Sjef Verbeek, Fons A. J. van de Loo, Marije I. Koenders, Peter M. van der Kraan, Arjen B. Blom, Martijn H. J. van den Bosch, Peter L. E. M. van Lent

**Affiliations:** 1 Experimental Rheumatology, Radboud university medical center, Geert Grooteplein Zuid 28, 6525 GA Nijmegen, the Netherlands; 2 Institute of Immunology, University of Münster, Münster, Germany; 3 Human Genetics, Leiden University Medical Center, Leiden, the Netherlands

**Keywords:** Experimental arthritis, Bone erosion, Osteoclast, Immune complexes, FcγRs, Neutrophils, S100A8

## Abstract

**Background:**

Osteoclast-mediated bone erosion is a central feature of rheumatoid arthritis (RA). Immune complexes, present in a large percentage of patients, bind to Fcγ receptors (FcγRs), thereby modulating the activity of immune cells. In this study, we investigated the contribution of FcγRs, and FcγRIV in particular, during antigen-induced arthritis (AIA).

**Methods:**

AIA was induced in knee joints of wild-type (WT), FcγRI,II,III^−/−^, and FcγRI,II,III,IV^−/−^ mice. Bone destruction, numbers of tartrate-resistant acid phosphatase-positive (TRAP^+^) osteoclasts, and inflammation were evaluated using histology; expression of the macrophage marker F4/80, neutrophil marker NIMPR14, and alarmin S100A8 was evaluated using immunohistochemistry. The percentage of osteoclast precursors in the bone marrow was determined using flow cytometry. In vitro osteoclastogenesis was evaluated with TRAP staining, and gene expression was assessed using real-time PCR.

**Results:**

FcγRI,II,III,IV^−/−^ mice showed decreased bone erosion compared with WT mice during AIA, whereas both the humoral and cellular immune responses against methylated bovine serum albumin were not impaired in FcγRI,II,III,IV^−/−^ mice. The percentage of osteoclast precursors in the bone marrow of arthritic mice and their ability to differentiate into osteoclasts in vitro were comparable between FcγRI,II,III,IV^−/−^ and WT mice. In line with these observations, numbers of TRAP^+^ osteoclasts on the bone surface during AIA were comparable between the two groups. Inflammation, a process that strongly activates osteoclast activity, was reduced in FcγRI,II,III,IV^−/−^ mice, and of note, mainly decreased numbers of neutrophils were present in the joint. In contrast to FcγRI,II,III,IV^−/−^ mice, AIA induction in knee joints of FcγRI,II,III^−/−^ mice resulted in increased bone erosion, inflammation, and numbers of neutrophils, suggesting a crucial role for FcγRIV in the joint pathology by the recruitment of neutrophils. Finally, significant correlations were found between bone erosion and the number of neutrophils present in the joint as well as between bone erosion and the number of S100A8-positive cells, with S100A8 being an alarmin strongly produced by neutrophils that stimulates osteoclast resorbing activity.

**Conclusions:**

FcγRs play a crucial role in the development of bone erosion during AIA by inducing inflammation. In particular, FcγRIV mediates bone erosion in AIA by inducing the influx of S100A8/A9-producing neutrophils into the arthritic joint.

**Electronic supplementary material:**

The online version of this article (10.1186/s13075-018-1584-1) contains supplementary material, which is available to authorized users.

## Background

Rheumatoid arthritis (RA) is a chronic and systemic autoimmune disease that primarily affects the joints [1]. Along with inflammation, excessive bone erosion is one of the central hallmarks of this disease [2, 3]. Next to generalized osteoporosis, severe focal bone erosions are observed at the interface between the inflamed synovium and the bone. Osteoclasts, which differentiate from myeloid precursor cells under the influence of macrophage colony-stimulating factor (M-CSF) and receptor activator of nuclear factor-κB ligand (RANKL), are the cells responsible for this deleterious process [4–6]. Therefore, a deeper understanding of how the inflammatory response increases bone erosion in this disease is likely to be helpful in identifying new therapeutic targets.

Autoantibodies such as rheumatoid factor (RF) and anticitrullinated protein antibodies are present in the serum and synovial fluid of a large percentage of patients with RA [1]. Of note, in patients with RA the presence of autoantibodies predates disease onset and correlates with disease progression and severity [7–9]. Immunoglobulin G (IgG) antibodies can form immune complexes (ICs) with their cognate antigens and subsequently bind to Fcγ receptors (FcγRs), thereby modulating the activity of the FcγR-bearing immune cells [10].

In mice, four different FcγRs have been identified, of which the activating FcγRI, FcγRIII, and FcγRIV stimulate the cell via the activation motif immunoreceptor tyrosine-based activation motif (ITAM), leading to effector functions such as phagocytosis, antigen presentation, and cytokine secretion. In contrast, FcγRIIb is an inhibitory receptor, and its intracellular domain contains an immunoreceptor tyrosine-based inhibitory motif (ITIM) that counteracts the signaling of the activating FcγRs [10–12]. Alterations in the expression of FcγRs have been described in circulating monocytes and synovial tissue of patients with RA, suggesting their involvement in the pathogenesis of RA [13–18]. Moreover, the crucial pathogenic role of FcγRs has been proven in a multitude of experimental arthritis models, such as collagen-induced arthritis (CIA), glucose-6-phosphate isomerase-induced arthritis, collagen type II antibody-induced arthritis, the K/B×N serum transfer model, IC arthritis, and antigen-induced arthritis (AIA) models. Overall, despite some differences between the various experimental models, activating FcγRs stimulate innate immune cells, leading to deleterious effects. On the contrary, FcγRIIb induces negative feedback in the production of autoantibodies, thereby protecting the joint from the development and progression of the disease [19–28]. However, the function of the various FcγRs and their exact mechanism of action in the modulation of bone erosion remain to be elucidated.

In the AIA experimental RA model, the injection of methylated bovine serum albumin (mBSA) into the knee joints of previously immunized mice results in a strong local accumulation of ICs that, via activation of the immune system, are responsible for the degradation of both bone and cartilage. In previous studies using this model, we determined the relationship between synovial inflammation and bone destruction using knockout mouse strains for various (combinations of) FcγRs. We found that there was a link between FcγR-mediated inflammation and bone erosion [29]. Whereas FcγRs are expressed on osteoclasts and may thus be involved in their differentiation and activation, a central role in IC-mediated inflammation has been attributed to the FcγR-mediated activation of macrophages during AIA [30]. Their IC-mediated activation leads to the production of a plethora of mediators, such as chemotactic factors, responsible for the recruitment of, among others, neutrophils into the joint. However, which FcγR is particularly involved in regulating this cell influx and which cell is dominant in mediating bone destruction is still a matter of debate.

The importance of neutrophils in arthritis development has been shown in the K/B×N serum transfer experimental RA model, in which depletion of neutrophils leads to complete protection from disease development [31]. In agreement with this finding, high numbers of neutrophils are present in the joints of patients with active RA [32, 33]. Two factors produced by neutrophils in high quantities are the alarmins S100A8 and S100A9, which make up roughly 40% of all cytosolic proteins [34]. S100A8/A9 are small calcium-binding proteins that, upon cell stress, are released into the extracellular environment, where they function as potent inducers of the immune system [35, 36]. High levels of S100A8/A9 are present in the synovial fluid of patients with RA [37, 38]. Moreover, it has been shown that S100A8 is able to directly stimulate osteoclast activity via TLR4, suggesting a possible mechanism through which the IC-activated innate immunity can regulate bone erosion in RA [39].

In the present work, we investigated the involvement of FcγRs, and of FcγRIV in particular, in the regulation of osteoclast-mediated bone resorption. We induced AIA in mice deficient in all four FcγRs (FcγRI,II,III,IV^−/−^ mice) and in their wild-type (WT) controls. The role of FcγRIV in particular was studied by comparing the development of AIA in FcγRI,II,III,IV^−/−^ and FcγRI,II,III^−/−^mice.

## Methods

### Animals

FcγRI,II,III,IV^−/−^ mice in a C57BL/6 background were developed by Dr. S. Verbeek (Leiden University Medical Center, Leiden, the Netherlands) (Dr.J.Sjef Verbeek personal communication, January 2016). Control C57BL/6 mice were purchased from Janvier Labs (Le Genest Saint Isle, France). FcγRI,II,III^−/−^ mice and their controls were generated as previously described [29]. Mice were housed under standard conditions in filter-top cages and fed a standard diet with food and tap water ad libitum. All animal studies were carried out according to the Dutch law and approved by the local animal experimentation committee (RU-DEC 2012-209).

### Induction of AIA

Mice were immunized with 100 μg of mBSA (Sigma-Aldrich, St. Louis, MO, USA) emulsified in complete Freund’s adjuvant (CFA; Difco Laboratories, Detroit, MI, USA). Heat-killed *Bordetella pertussis* was administrated intraperitoneally as an additional adjuvant. One week later, two subcutaneous injections in the neck region with a total of 50 μg of mBSA/CFA were administered as a booster. Three weeks after the immunization, arthritis was induced in both knee joints by intra-articular injection of 60 μg of mBSA in 6 μl of saline.

### Serum collection and antibody titer determination in serum

At day 7 and day 21 after AIA induction, blood was drawn from the retro-orbital plexus in MiniCollect tubes (Greiner Bio-One, Monroe, NC, USA), and subsequently serum was obtained by centrifugation. Anti-mBSA-specific antibodies (total IgG, IgG1, IgG2a, IgG2b) were measured in sera with an enzyme-linked immunosorbent assay. mBSA was coated on plates (Nunc; Thermo Fisher Scientific, Rochester, NY, USA) at a concentration of 100 μg/ml. Antibody concentrations were assessed by twofold serial dilution of the sera, followed by detection of bound mouse IgG with peroxidase-conjugated rabbit antimouse IgG (SouthernBiotech, Birmingham, AL, USA). 5-Aminosalicylic acid was used as a substrate. Absorbance was measured at 450 nm. Antibody titers were determined at 50% of the maximum absorption.

### Lymphocyte stimulation test

Spleens were collected from mice at day 21 after AIA induction and homogenized through a cell strainer. Erythrocytes were lysed with lysis buffer (155 mM NH_4_Cl, 12 mM KHCO_3_, 0.1 mM ethylenediaminetetraacetic acid, pH 7.3). Cells were seeded into flasks, and after 1 hour at 37 °C, nonadherent cells were harvested and seeded into 96-well plates (1 × 10^5^ cells/well). mBSA was added at final concentrations of 50, 25, 12.5, 6.25, 3.12, and 1.56 μg/ml. Concanavalin A and ovalbumin were used as positive and negative controls, respectively. Cultures were maintained for 4 days. [3H]Thymidine was added for the last 16 hours of culture, and its incorporation was determined as a measure of T-cell proliferation.

### Histological analysis

Total knee joints were isolated, fixed in 4% phosphate-buffered formalin, decalcified in 5% formic acid, embedded in paraffin, and 7-μm coronal sections of various depths of the joint were made. Sections were stained with H&E for histological analysis. Inflammation (infiltrate and exudate) was arbitrarily scored on a scale from 0 (no inflammation) to 3 (severe inflammation). Bone destruction was evaluated in 13 well-defined areas of the knee joint (as depicted in the scheme in Additional file 1a) with a score ranging from 0 (no erosion) to 3 (connection between joint cavity and bone marrow). For the evaluation of proteoglycan (PG) depletion as a measure of cartilage destruction, joint sections were stained with Safranin O and Fast Green. PG depletion was evaluated at both the patellofemoral and the tibiofemoral areas as the amount of red staining present, using an arbitrary score ranging from 0 (absence of PG depletion) to 3 (complete PG depletion). For quantification of the number of osteoclasts, total knee joint sections were stained for tartrate-resistant acid phosphatase (TRAP), using the Leukocyte Acid Phosphatase Kit (Sigma-Aldrich) according to the manufacturer’s protocol. The number of TRAP^+^ cells present along the external bone surface was counted. For quantification of periarticular bone, the percentage of noncartilage collagenous tissue (blue staining) in the complete femur and tibia of joint sections stained with Safranin O and Fast Green was quantified using Leica Application Suite software (Leica Microsystems, Buffalo Grove, IL, USA).

### Immunohistochemistry

To visualize S100A8-, NIMPR14-, and F4/80-expressing cells, knee joint sections were incubated with specific primary antibodies against S100A8 (made in our facilities), NIMPR14 (kindly provided by Dr. M. Strath, London, UK) and F4/80 (Thermo Fisher Scientific). Afterward, sections were incubated with horseradish peroxidase-conjugated or biotinylated secondary antibodies followed by avidin-biotin complex peroxidase (VECTASTAIN Elite Kit; Vector Laboratories, Burlingame, CA, USA). Antibody binding was visualized using diaminobenzidine. S100A8 staining was arbitrarily scored using a scale from 0 to 3. For quantification of NIMPR14- and F4/80-positive cells, pictures (original magnification ×100) of five specific areas of the joint were taken (two in the area adjacent to the patella and two in the area adjacent to the medial and lateral femur for the evaluation of infiltrate, and one in the area of the joint cavity between the patella and femur for evaluation of the exudate). The amount of cells in the infiltrate was measured as the positive area above a fixed threshold using Leica Application Suite software (Leica Microsystems). The number of positive cells in the exudate was counted using the cell counter plugin of ImageJ software (National Institutes of Health, Bethesda, MD, USA).

### Flow cytometric analysis

Bone marrow was isolated from femurs and tibias of mice by flushing the marrow cavity with medium and passing the cell suspension through a cell strainer. After lysis of erythrocytes, bone marrow cells were incubated with Fc-blocking antibody (BD Pharmingen antimouse CD16/CD32, clone 2.4G2; BD Biosciences, San Jose, CA, USA), followed by staining with the following mix of antibodies: CD11b-fluorescein isothiocyanate, CD90.2-phycoerythrin (PE), CD45R/B220-PE, CD49b-PE, NK1.1-PE, Ly6G-PE, Ly6C-allophycocyanin-cyanine 7 (all BD Biosciences). Samples were acquired with a CyAn flow cytometer (Beckman Coulter Life Sciences, Indianapolis, IN, USA), and data analysis was performed with Kaluza Analysis Software (Beckman Coulter Life Sciences). The gating strategy we used is depicted in Additional file 2.

### Bone marrow-derived osteoclast differentiation

Bone marrow was isolated from femurs and tibias of mice. Total bone marrow cells were seeded into 96-well plates at a density of 10^5^ cells/well in 150 μl of α-minimum essential medium (Thermo Fisher Scientific), supplemented with 5% FCS, penicillin/streptomycin, 30 ng/ml recombinant mouse (rm)M-CSF, and 20 ng/ml rmRANKL (R&D Systems, Minneapolis, MN, USA). Culture medium was refreshed after 3 days.

### Measurement of TRAP activity in the supernatants

Cell supernatants were collected after 5 days of differentiation, and TRAP activity was measured with a colorimetric assay. In short, *p*-nitrophenyl phosphate (New England Biolabs, Ipswich, MA, USA) was diluted in buffer containing 420 mM acetic acid (Sigma-Aldrich) and 160 mM tartrate solution (Merck, Kenilworth, NJ, USA) and added 1:1 to culture supernatant. After 1 hour, the reaction was stopped with 0.5 M NaOH (Sigma-Aldrich), and the absorbance at 405 nm was determined using a spectrophotometric plate reader (Bio-Rad Laboratories, Hercules, CA, USA).

### RNA isolation and qRT-PCR

Well-defined synovial samples were isolated from the inflamed murine knee joints as previously described [40]. Tissue samples were homogenized using the MagNA Lyser Instrument (Roche Diagnostics, Indianapolis, IN, USA). Total RNA was isolated using the RNeasy Fibrous Tissue Mini Kit (Qiagen, Hilden, Germany). RNA from osteoclast cultures was isolated with TRIzol reagent (Sigma-Aldrich). RNA was subsequently reverse-transcribed into complementary DNA. qRT-PCR was performed using the Applied Biosystems StepOnePlus RT-PCR System (Thermo Fisher Scientific, Foster City, CA, USA). Primer sequences are listed in Table 1 (primers obtained from Biolegio, Nijmegen, the Netherlands). Glyceraldehyde 3-phosphate dehydrogenase (*Gapdh*) was used as the reference gene. Samples were normalized for the expression of *Gapdh* by calculating the comparative threshold: −Δ*C*_t_ = − (*C*_t_ gene of interest − *C*_t_ *Gapdh*).

**Table 1:** ᅟ

Gene	Primer sequences (5′-3′)
*Gapdh*	Forward: GGCAAATTCAACGGCACA Reverse: GTTAGTGGGGTCTCGCTCCTG
*Nfatc1*	Forward: ATGCGAGCCATCATCGA Reverse: GGGATGTGAACTCGGAAGAC
*Acp5*	Forward: GACAAGAGGTTCCAGGAG ACC Reverse: GGGCTGGGGAAGTTCCAG
*Calcr*	Forward: CGTTCTTTATTACCTGGCTCTTGTG Reverse: TCTGGCAGCTAAGGTTCTTGA AA
*Mmp9*	Forward: GGAACTCACACGACATCTTCCA Reverse: GAAACTCACACGCCAGAAGAATTT
*Ca2*	Forward: GCTGCAGAGCTTCACTTGGT Reverse: AAACAGCCAATCCATCCGGT
*Oscar*	Forward: TGGTCATCAGTTTCGAAGGTTCT Reverse: CAGCCCCAAACGGATGAG
*Dcstamp*	Forward: TGTATCGGC TCATCTCCTCCAT Reverse: GACTCCTTGGGTTCCTTGCTT
*Clcn7*	Forward: AGCCTGGACTATGACAACAGC Reverse: GGAAAGCCGTGTGGTTGATT
*Ctsk*	Forward: GAAGCAGTATAACAGCAAGGTGGAT Reverse: TGTCTCCCAAGTGGTTCATGG

### Statistical analysis

Statistical differences between two groups were calculated using Student’s *t* test for parametric variables (messenger RNA [mRNA] expression, IgG titer) or the Mann-Whitney *U* test for nonparametric variables (lymphocyte stimulation test/arbitrary score of inflammation, PG depletion, and S100A8 staining/percentage of osteoclast precursors/number of TRAP-positive cells in vivo/TRAP activity in the supernatant/quantification of NIMPR14- and F4/80-positive cells/percentage of noncartilage calcified tissue). For comparison of multiple groups in the quantification of in vivo bone erosion and TRAP^+^ cells after in vitro differentiation, two-way analysis of variance was used. Spearman’s rank correlation coefficients (*r*_S_) were calculated for correlation analysis. All analyses were performed using Prism version 5.03 software (GraphPad Software, La Jolla, CA, USA), and *P* values less than 0.05 were considered significant.

## Results

### Decreased bone erosion in FcγRI,II,III,IV^−/−^ mice compared with WT controls

**Fig. 1:**
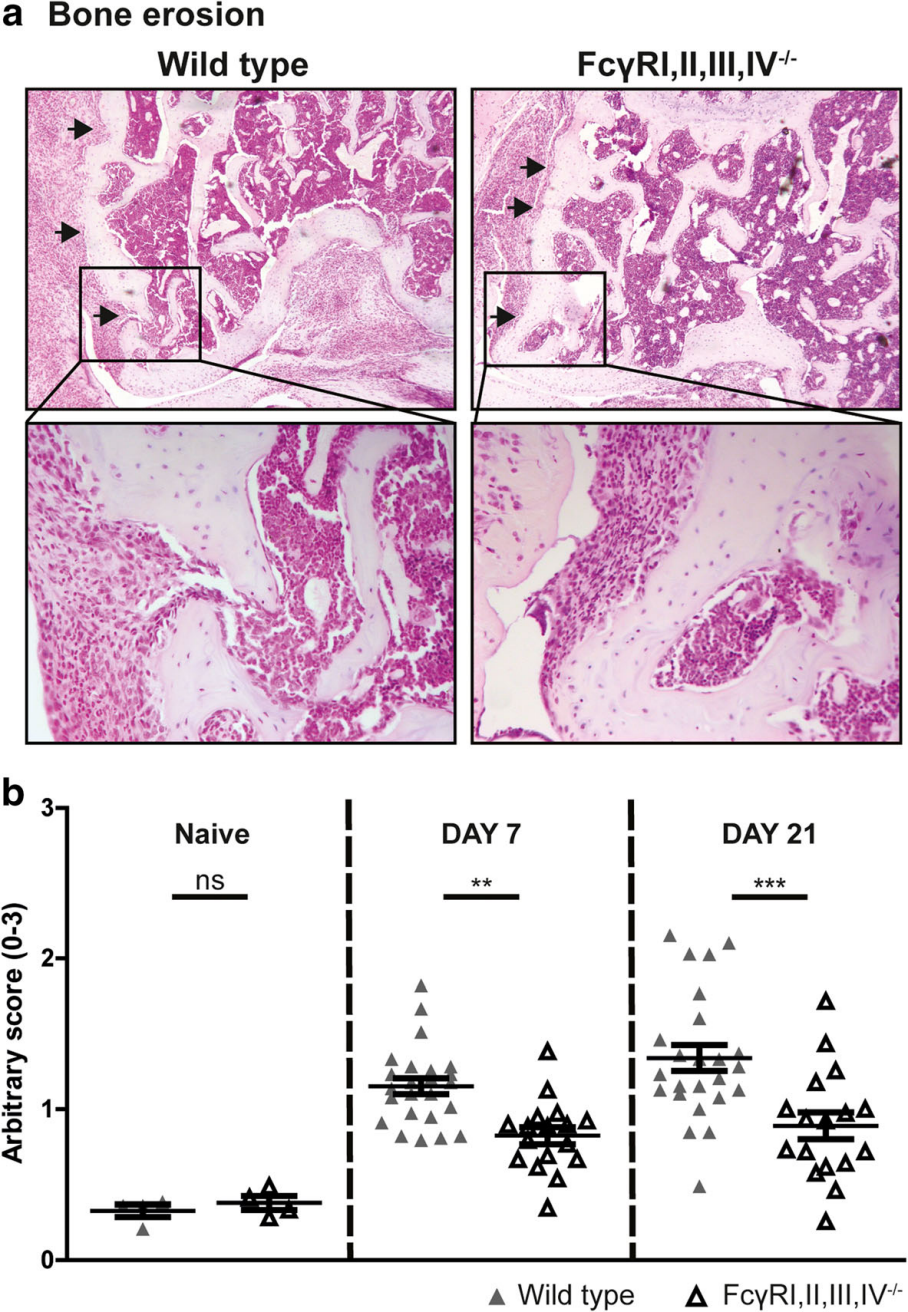
Fcγ receptor (FcγR)I,II,III,IV^−/−^ mice have decreased bone erosion compared with wild-type (WT) control mice. **a** Bone erosion (*black arrows*) is present in the joints of FcγRI,II,III,IV^−/−^ and WT mice after induction of antigen-induced arthritis (AIA), as determined by using H&E-stained sections. Original magnification ×50 and ×200. **b** Quantification of bone erosion showed significantly decreased resorption in the joints of FcγRI,II,III,IV^−/−^ mice compared with their WT controls (*n* = 17 and 24 joints per group respectively) at both 7 and 21 days after AIA induction. No basal differences in bone erosion could be observed in the joints of naive mice. Scatterplots are shown, with *horizontal* and *vertical lines* representing mean ± SEM values. *ns* Not significant. ** *P* < 0.01, *** *P* < 0.001 versus WT controls

First, to determine the effect of the absence of FcγRs on bone resorption during experimental arthritis, we scored bone erosion after induction of AIA in FcγRI,II,III,IV^−/−^ mice and their WT controls. At both day 7 and day 21 after AIA induction, we observed significantly decreased bone erosion in the FcγRI,II,III,IV^−/−^ mice as compared with their WT controls, highlighting the importance of FcγRs in this process (Fig. 1). However, although the bone erosion was significantly decreased in the FcγRI,II,III,IV^−/−^ mice compared with their WT controls, scores were not reduced to the same level of in naive mice. To investigate a possible basal phenotype due to the absence of FcγRs, we determined the bone erosion in knee joints of WT and FcγRI,II,III,IV^−/−^ mice without AIA. This showed comparable basal levels of bone erosion in these naive knee joints (Fig. 1b). In addition, no differences were found in the surface area of noncartilage collagenous tissue present in the femur and tibia (Additional file 1b). Together, these findings suggest that the decreased bone resorption that was observed in the FcγRI,II,III,IV^−/−^ mice after induction of experimental arthritis could not be explained by an underlying basal bone phenotype. As an additional readout of joint damage, we evaluated PG depletion as a measure of inflammation-induced cartilage destruction at the patellofemoral and tibiofemoral regions. In line with the decreased bone damage, we observed decreased PG depletion in FcγRI,II,III,IV^−/−^ mice compared with their WT controls at day 7 and day 21 after AIA induction, particularly in the tibiofemoral area (Additional file 1c).

### Comparable immune response against mBSA in WT and FcγRI,II,III,IV^−/−^ mice

**Fig. 2:**
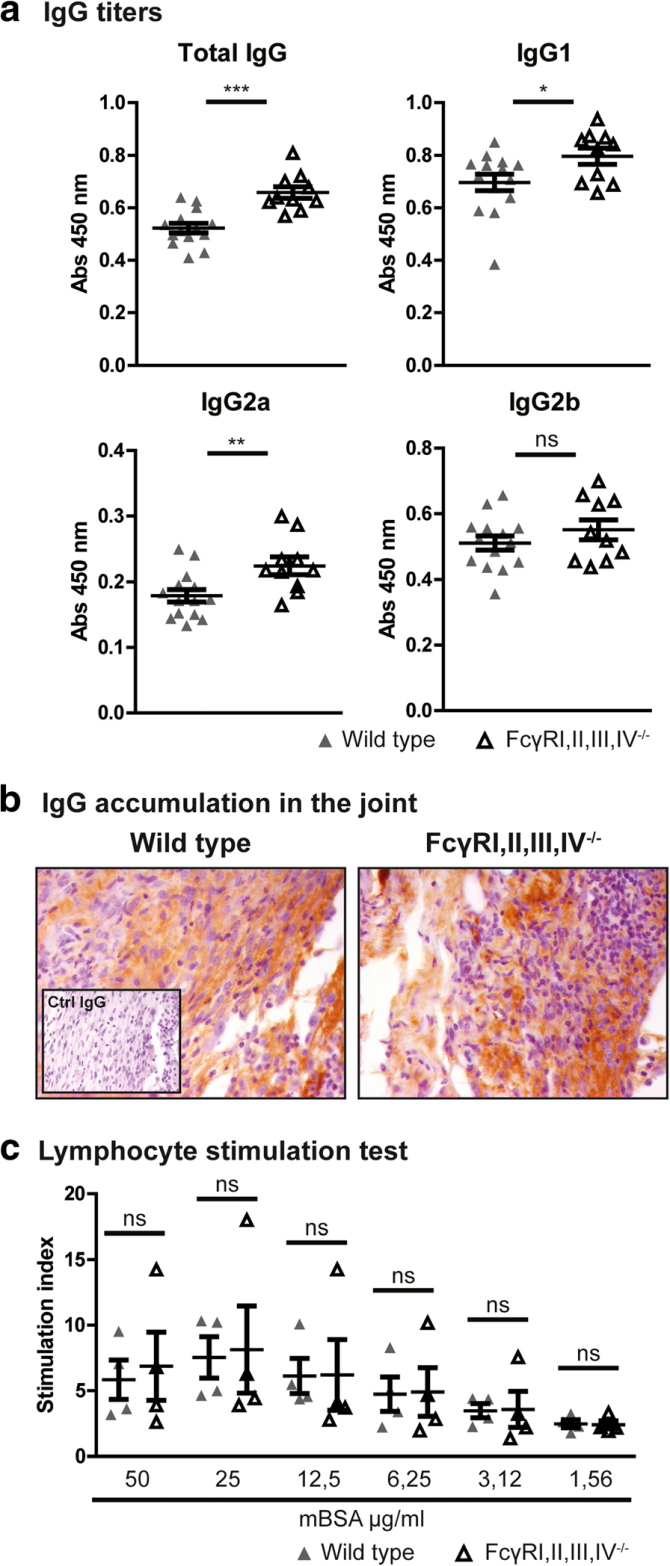
Fcγ receptor (FcγR)I,II,III,IV deficiency does not impair the humoral and cellular immune responses against methylated bovine serum albumin (mBSA). **a** Significantly increased anti-mBSA total immunoglobulin G (IgG), IgG1, and Ig2a titers were measured in the serum of FcγRI,II,III,IV^−/−^ mice compared with those of wild-type (WT) mice, whereas IgG2b titers were not significantly different (WT, *n* = 14; FcγRI,II,III,IV^−/−^, *n* = 10; combined from day 7 and day 21). **b** Accumulation of IgG was present in the knee joints of both WT and FcγRI,II,III,IV^−/−^ mice 7 days after induction of antigen-induced arthritis as determined by immunolocalization. Original magnification ×400. **c** The cellular anti-mBSA immune response, as determined by T-cell proliferation, was comparable between WT and FcγRI,II,III,IV^−/−^ mice. Results are expressed as stimulation index (ratio of stimulation with/without antigen) (*n* = 4 mice/group). Scatterplots are shown, with *horizontal* and *vertical lines* representing mean ± SEM values. *ns* Not significant. * *P* < 0.05, ** *P* < 0.01, *** *P* < 0.001

Because the induction of the AIA model is highly dependent on the immune response against the mBSA antigen, we set out to determine whether the humoral and cellular immune responses against mBSA were affected by the absence of FcγRI,II,III,IV. Therefore, we determined anti-mBSA IgG titers in the serum of mice with AIA. We found increased titers of total IgG, IgG1, and IgG2a, but a comparable titer of IgG2b, in FcγRI,II,III,IV^−/−^ mice as compared with WT mice (Fig. 2a). Moreover, using immunostaining against IgG in the joint, we showed IgG accumulation in both WT and FcγRI,II,III,IV^−/−^ mice (Fig. 2b). Finally, we observed comparable mBSA-induced proliferation of T cells obtained from FcγRI,II,III,IV^−/−^ and WT mice (Fig. 2c).

### Absence of Fcγ receptors does not affect the number of osteoclast precursors

**Fig. 3:**
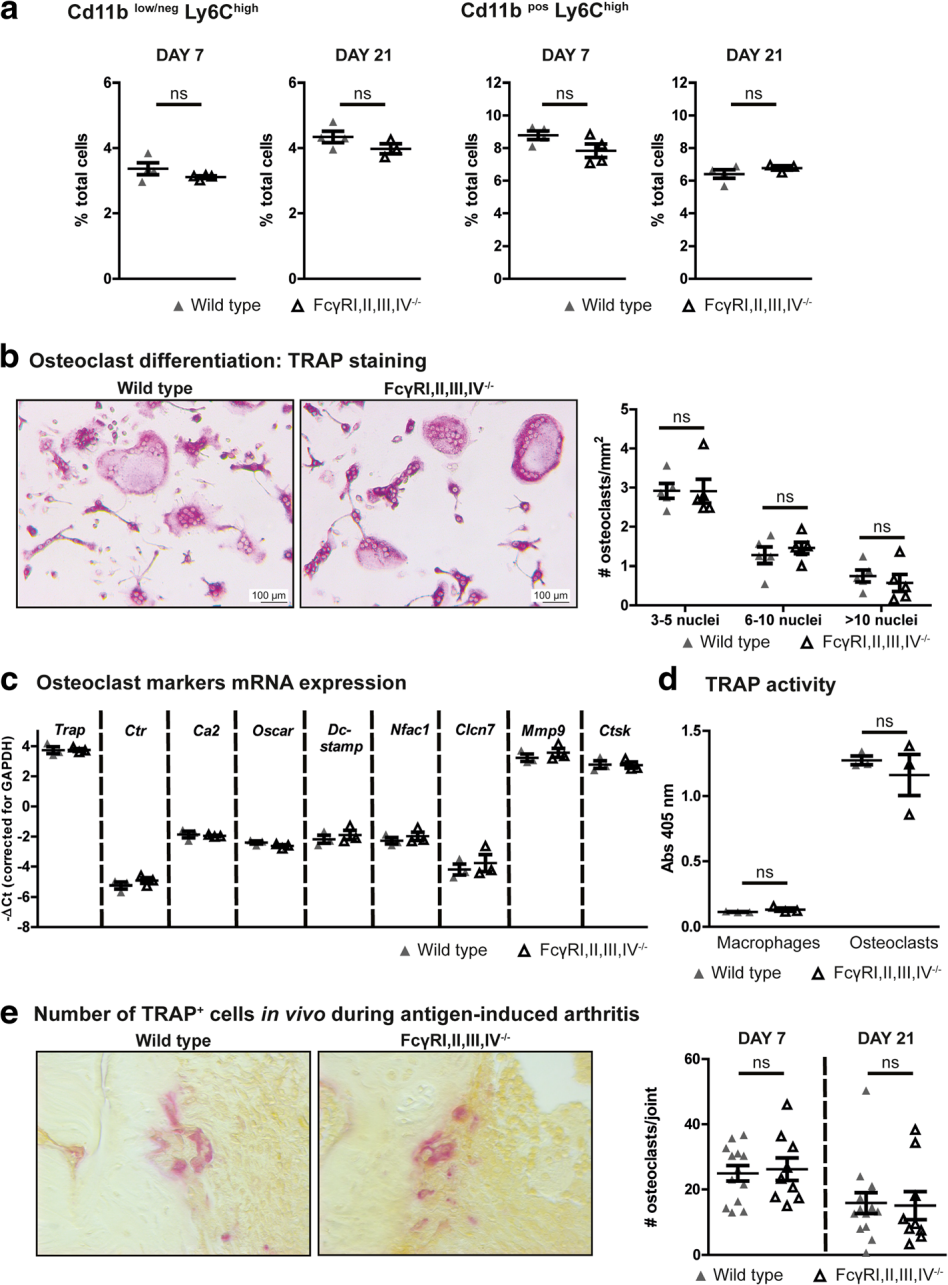
Absence of Fcγ receptor (FcγR)I,II,III,IV does not affect the percentage of osteoclast precursors and their osteoclastogenic potential. **a** Comparable percentages of CD11b^pos^Ly6C^high^ and CD11b^low/neg^Ly6C^high^ osteoclast precursors were present in the bone marrow of wild-type (WT) and FcγRI,II,III,IV^−/−^ mice at days 7 and 21 of antigen-induced arthritis (AIA) (*n* = 3 or 4 mice/group). **b** Images of tartrate-resistant acid phosphatase (TRAP) staining of osteoclasts after 5 days of in vitro differentiation. Quantification showed comparable numbers of osteoclasts obtained from WT and FcγRI,II,III,IV^−/−^ bone marrow cells (*n* = 5 mice/group). **c** Moreover, comparable messenger RNA expression levels of various osteoclast markers were determined in WT and FcγRI,II,III,IV^−/−^ osteoclasts (*n* = 3 mice/group). **d** Finally, TRAP activity in the culture supernatant of WT and FcγR I,II,III,IV^−/−^ macrophages and osteoclasts was comparable (*n* = 3 mice/group). **e** Representative photomicrographs of TRAP staining and quantification of the number of positive cells along the bone surface in total knee joint sections of WT and FcγRI,II,III,IV^−/−^ mice at days 7 and 21 after induction of AIA (*n* = 14 and *n* = 10 per time point for WT and FcγRI,II,III,IV^−/−^ mice, respectively). Scatterplots are shown, with *horizontal* and *vertical lines* representing mean ± SEM values. *ns* Not significant

In the next set of experiments, we aimed at elucidating the mechanism underlying the decreased bone erosion observed in the absence of FcγRs. We first determined whether the FcγRI,II,III,IV^−/−^ mice had a different percentage of osteoclast precursors in their bone marrow after induction of AIA. The percentage of CD11b^pos^Ly6C^high^ and CD11b^low/neg^Ly6C^high^ cells, both of which have been shown to differentiate into osteoclasts [41, 42], were comparable between FcγRI,II,III,IV^−/−^ and WT mice (Fig. 3a). These data suggest that the observed decrease in bone erosion does not originate from differences in osteoclast precursor populations.

### Absence of Fcγ receptors does not affect the osteoclastogenic potential and the number of osteoclasts on the bone surface during AIA

Next, we determined whether the osteoclast progenitors from FcγRI,II,III,IV^−/−^ mice have the same osteoclastogenic potential as WT cells. After in vitro differentiation of bone marrow cells into osteoclasts with M-CSF and RANKL, we observed comparable numbers of multinucleated TRAP^+^ cells in the cultures with WT and FcγRI,II,III,IV^−/−^ cells (Fig. 3b). In agreement with this finding, we found comparable mRNA expression levels of key osteoclast differentiation markers, such as nuclear factor of activated T-cells, cytoplasmic 1 (*Nfatc1*), *TRAP*, dendritic cell-specific transmembrane protein (*Dcstamp*), calcitonin receptor (*Ctr*), and osteoclast-associated immunoglobulin-like receptor (*Oscar*), as well as comparable mRNA expression levels of activation markers, such as chloride channel 7 (*Clcn7*), carbonic anhydrase II (*Ca2*), matrix metallopeptidase 9 (*Mmp9*), and cathepsin K (*Ctsk*) (Fig. 3c). Moreover, the TRAP enzyme activity as measured in the supernatant of the osteoclast cultures was comparable (Fig. 3d). Together, these findings show that the absence of FcγRs does not affect the osteoclastogenic potential of precursor cells. Finally, because in vivo osteoclastogenesis is a complex process that can be influenced by many factors, we determined the number of osteoclasts on the bone surface of FcγRI,II,III,IV^−/−^ and WT mice during AIA using TRAP staining. Interestingly, in line with their comparable osteoclastogenic potential in vitro, the number of TRAP^+^ cells along the bone surface did not differ between FcγRI,II,III,IV^−/−^ and WT mice both at day 7 and day 21 after AIA induction (Fig. 3e).

### FcγRs differentially regulate the influx of neutrophils present in the joint

**Fig. 4:**
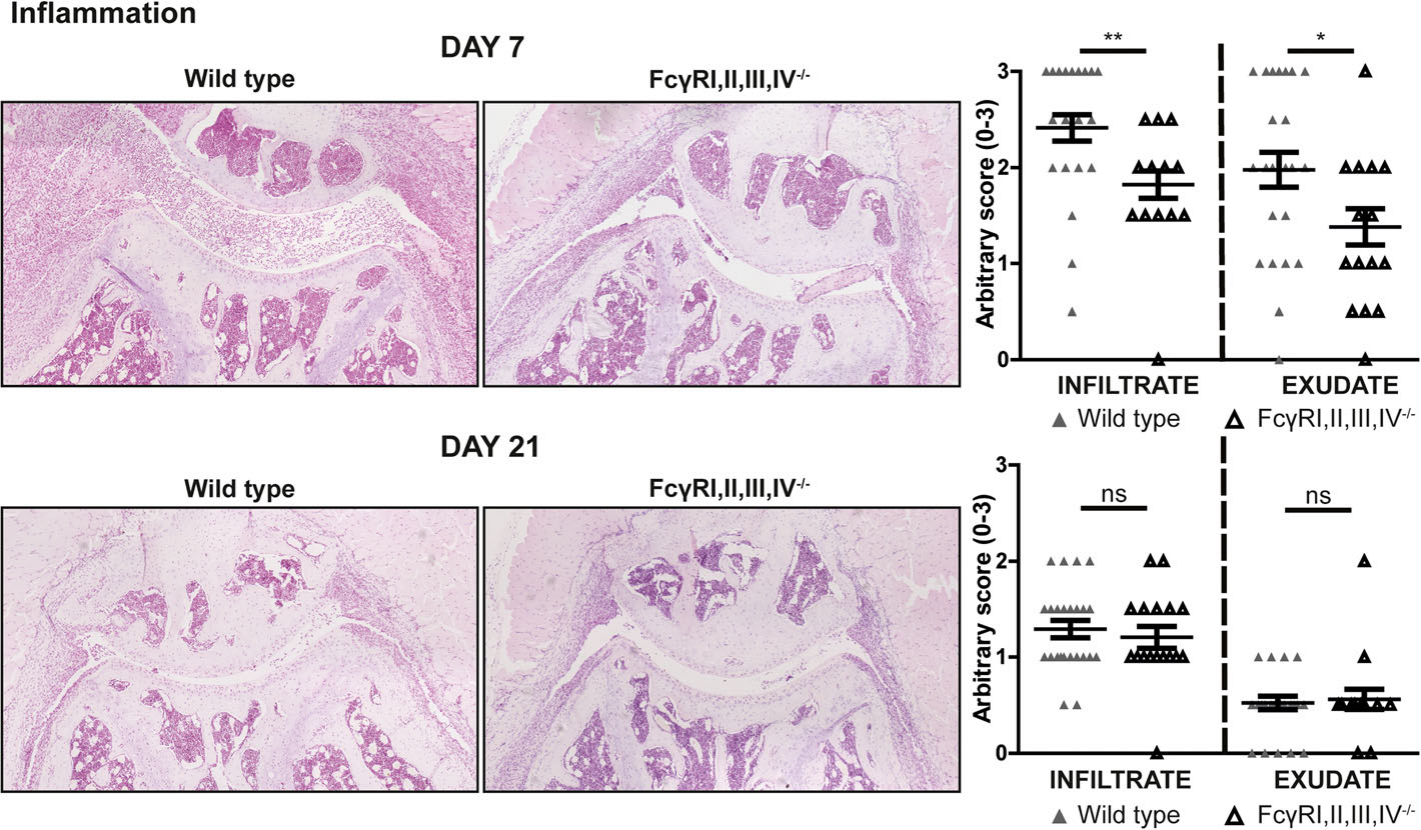
Fcγ receptor (FcγR)I,II,III,IV^−/−^ mice have decreased inflammation in arthritic joints. Photomicrographs of H&E staining showing the infiltrate and exudate in knee joints of wild-type (WT) and FcγRI,II,III,IV^−/−^ mice at days 7 and 21 of antigen-induced arthritis (AIA). Original magnification ×100. Quantification showed decreased infiltrate and exudate in the knee joints of FcγRI,II,III,IV^−/−^ mice compared with WT controls at day 7 of AIA. In contrast, no differences were observed at day 21 (*n* = 24 and *n* = 17 joints per time point for WT and FcγRI,II,III,IV^−/−^ mice, respectively). Scatterplots are shown, with *horizontal* and *vertical lines* representing mean ± SEM values. *ns* Not significant. * *P* < 0.05, ** *P* < 0.01

**Fig. 5:**
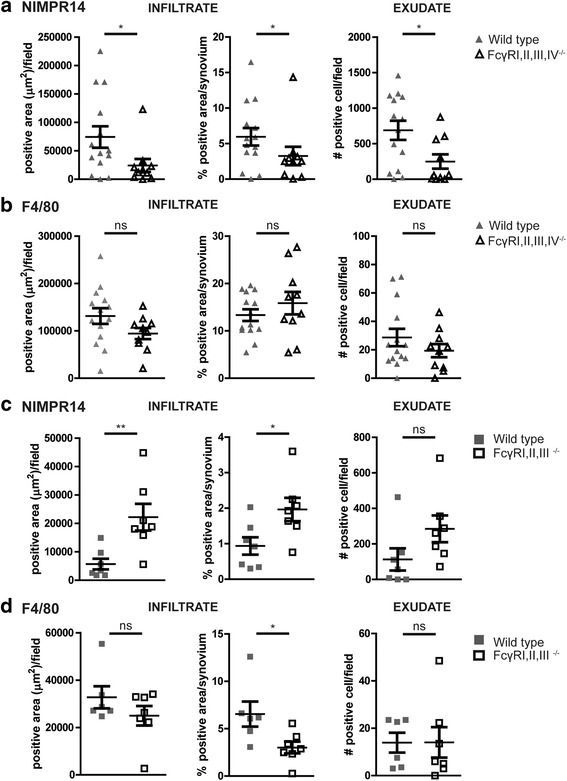
Prominent role for Fcγ receptor (FcγR)IV in regulating the influx of neutrophils into the joint. **a** NIMPR14-positive area, percentage of NIMPR14-positive area in the infiltrate, and number of NIMPR14-positive cells in the exudate are decreased in FcγRI,II,III,IV^−/−^ mice compared with wild-type (WT) mice in day 7 antigen-induced arthritis (AIA). **b** Comparable numbers of F4/80-positive cells are present in the joints of FcγRI,II,III,IV^−/−^ mice compared with their WT controls at day 7 AIA. **c** In contrast to FcγRI,II,III,IV^−/−^ mice, the total NIMPR14-positive area, percentage of NIMPR14-positive area in the infiltrate, and number of NIMPR14-positive cells in the exudate are increased in FcγR I,II,III^−/−^ mice. **d** Comparable numbers and reduced percentage of F4/80-positive cells in the joint of FcγR I,II,III^−/−^ mice compared with their WT controls at day 7 of AIA. Scatterplots are shown, with *horizontal* and *vertical lines* representing mean ± SEM values of at least six mice per group. *ns* Not significant. * *P* < 0.05, ** *P* < 0.01

Because proinflammatory cells and their products can strongly increase the resorbing activity of osteoclasts, we evaluated the severity of inflammation in the arthritic knee joints. The degree of both infiltrate and exudate was significantly decreased in the knee joints of FcγRI,II,III,IV^−/−^ mice at day 7 after AIA induction (Fig. 4). At day 21 after induction, the degree of inflammation was decreased in both strains, and no significant difference could be observed between FcγRI,II,III,IV^−/−^ and WT mice anymore. The early phase of inflammation (day 7) during AIA is particularly characterized by an abundant presence of neutrophils in the exudate and infiltrate in the knee joint. Interestingly, we observed significantly decreased numbers of NIMPR14-positive neutrophils in the exudate, and the NIMPR14-positive area in the infiltrate was significantly lower in FcγRI,II,III,IV^−/−^ mice than in WT mice (Fig. 5a). In contrast, numbers of F4/80-positive monocytes/macrophages in both the exudate and infiltrate were comparable between FcγRI,II,III,IV^−/−^ and WT mice (Fig. 5b), and a trend toward an increased percentage of F4/80 cells in the infiltrate of FcγRI,II,III,IV^−/−^ mice was observed. Previous data developed at our laboratory showed that in contrast to the decreased inflammation and bone resorption in FcγRI,II,III,IV^−/−^ mice that we describe in the present work, FcγRI,II,III^−/−^ mice showed increased bone resorption, together with more inflammation, compared with their WT controls after induction of AIA [29]. Of note, we observed a significant increase and a trend toward an increase in the numbers of neutrophils present in the infiltrate and exudate, respectively, of these FcγRI,II,III^−/−^ mice (Fig. 5c). However, in agreement with our findings in the FcγRI,II,III,IV^−/−^ mice, the number of F4/80-positive monocytes/macrophages cells was not significantly different between FcγRI,II,III^−/−^ and WT mice, and the percentage of F4/80-positive area was decreased in FcγRI,II,III^−/−^ mice (Fig. 5d). Representative photomicrographs are shown in Additional files 3 and 4. Together, these findings suggest that FcγRIV might be of particular importance in the recruitment of neutrophils into the arthritic joint and that these neutrophils likely contribute to the bone erosion process.

### Numbers of S100A8/A9-producing neutrophils strongly correlate with the amount of bone erosion

**Fig. 6:**
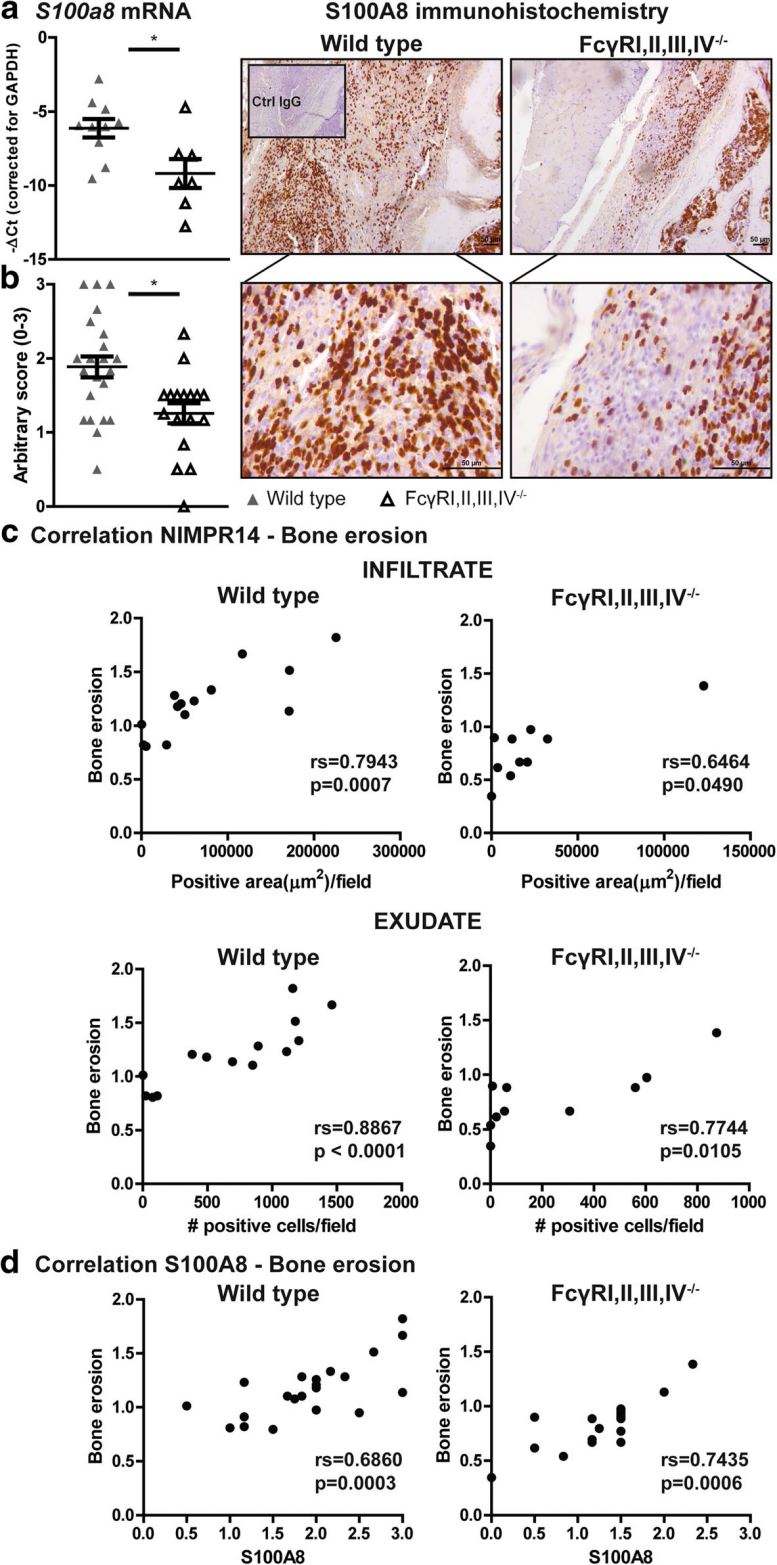
The number of S100A8-producing neutrophils correlates with the severity of bone erosion during antigen-induced arthritis. **a** *S100a8* messenger RNA levels in the synovium of Fcγ receptor (FcγR)I,II,III,IV^−/−^ mice are decreased compared with wild-type (WT) mice at day 7 of antigen-induced arthritis (AIA). **b** Representative photomicrographs show S100A8 staining in knee joint sections of WT and FcγRI,II,III,IV^−/−^ mice at day 7 of AIA. Original magnification ×100 and ×400. Quantification showed a decreased number of S100A8-positive cells in FcγRI,II,III,IV^−/−^ mice compared with their WT controls. **c** A significant correlation was observed between the number of neutrophils (NIMPR14-positive cells) in the infiltrate and exudate in the joint and bone erosion in both WT and FcγRI,II,III,IV^−/−^ mice at day 7 of AIA. **d** A significant correlation between the number of S100A8-positive cells in the joint and the severity of bone erosion in both WT and FcγRI,II,III,IV ^−/−^ mice at day 7 of AIA was found. * *P* < 0.05. rs = Spearman’s rank correlation coefficient

In line with the decreased numbers of neutrophils present in their joints, lower expression levels of S100A8, at both the mRNA and protein levels, were observed in FcγRI,II,III,IV^−/−^ than in WT mice (Fig. 6a and b). Moreover, in agreement with the higher numbers of neutrophils observed in the joints of FcγRI,II,III^−/−^ mice, increased expression of S100A8 was previously described by researchers at our laboratory [24]. Supporting our idea that the S100A8 produced by neutrophils plays an important role in the observed bone erosion, we found that the number of neutrophils in both the infiltrate and exudate, as well as their production of S100A8, strongly and significantly correlated with the severity of bone erosion (Fig. 6c and d).

## Discussion

In the present study, we show that FcγRs are crucially involved in bone erosion during AIA. Moreover, we show that the absence of all FcγRs does not affect the number of osteoclast precursors or their osteoclastogenic potential, but that it decreases the subsequent bone erosion during experimental arthritis via a reduction of inflammation. The comparison of development of AIA in FcγRI,II,III,IV^−/−^ and FcγRI,II,III^−/−^ mice suggested a possible crucial role of FcγRIV in mediating neutrophil inflammation during AIA.

We observed that mice lacking all FcγRs had decreased inflammation at day 7 and decreased bone erosion at both day 7 and day 21 after induction of the disease. This is in agreement with a previous study by Hobday and colleagues, which showed that FcγRI,II,III,IV^−/−^ mice were completely protected from serum-transferred arthritis at a macroscopic level [43]. However, in contrast to this serum transfer model, the AIA model is characterized by clear T-cell involvement, which is probably FcγR-independent. This might explain why we did not observe complete protection in FcγRI,II,III,IV^−/−^ mice. In another study, in line with what we observed in FcyRI,II,III,IV-/- mice, y-chain/FcyRIIb -/- mice lacking signaling of both activating and inhibitory FcγRs, as well as of other receptors using the γ-chain, were completely protected from development of CIA, although bone erosion data were not reported [44].

The induction of the AIA experimental RA model is highly dependent on the binding of mBSA/anti-mBSA-containing ICs to FcγRs, thereby potently activating the cell, and on the activation of T-cell responses against mBSA. However, we observed comparable T-cell responses against the mBSA antigen in FcγRI,II,III,IV^−/−^ and WT animals, which shows that their contribution to the induction of the model was not affected by the absence of the FcγRs. This is consistent with previous studies in which normal T-cell responses were found in the absence of either activating or inhibitory FcγRs after the induction of the AIA [27, 28]. Together, these findings suggest that the development of the T-cell immune response is FcγR-independent.

It has previously been shown that the absence of FcγRIIb often leads to increased IgG titers in mice owing to a lack of negative feedback on the production of IgGs by plasma cells, which results in enhanced stimulation of the immune response [28, 45]. In line with these findings, the FcγRI,II,III,IV^−/−^ mice used in this study, which also lack FcγRIIb, showed increased IgG titers (total IgG, IgG1, IgG2a) compared with their WT controls. Moreover, accumulation of IgG was present in the joints of FcγRI,II,III,IV^−/−^ mice. Together, these data showing a normal T-cell response against mBSA and increased IgG titers in FcγRI,II,III,IV^−/−^ mice suggest that the observed decrease in bone pathology cannot be the result of an insufficient immune response against mBSA after induction of the AIA model.

Osteoclasts are the main cells responsible for the degradation of bone tissue during RA. Although it is known that osteoclasts differentiate from bone marrow-derived myeloid precursors under the influence of M-CSF and RANKL, a strictly defined osteoclast precursor set has not yet been identified [4]. CD11b^pos^Ly6C^high^ bone marrow monocytes have been reported to differentiate into osteoclasts when stimulated with M-CSF and RANKL in vitro, and depletion of Ly6C^high^ cells in vivo results in decreased osteoclast formation and subsequent bone resorption in the K/B×N serum transfer model [41]. Moreover, Charles and colleagues recently identified the CD11b^low/neg^Ly6C^hi^ bone marrow population as having osteoclastogenic potential both in vitro and in vivo [42]. In the present study, we did not observe differences in the relative percentages of both the Ly6C^high^ and CD11b^low/neg^Ly6C^high^ osteoclast precursor populations between FcγRI,II,III,IV^−/−^ and WT mice, which allowed us to exclude the possibility that decreased bone erosion in the FcγRI,II,III,IV^−/−^ mice was merely the result of decreased numbers of osteoclast precursors.

Next to M-CSF and RANKL signaling, a costimulatory signal via the activation of the ITAM domain, which is present in the γ-chain and in DNAX activation protein of 12 kDa (DAP-12), is required for the activation of NFATc1, which is the transcription factor essential for osteoclast differentiation [46, 47]. Because activating FcγRI, FcγRIII, and FcγRIV are expressed by osteoclasts and are associated with the ITAM-containing γ-chain, they potentially can affect osteoclast differentiation. However, in the present study, we found that the absence of all four FcγRs does not impact the differentiation of osteoclasts from their precursors in vitro. Underlining our findings, Negishi-Koga and colleagues showed that FcγRIIb^−/−^/γ-chain^−/−^ cells, which lack signaling of both activating and inhibitory FcγRs, did not show alterations in osteoclast differentiation. However, varying results have previously been described concerning the effects of FcγRs on osteoclastogenesis. Surprisingly, Negishi-Koga and colleagues reported that FcγRIII^−/−^ mice have an osteoporotic phenotype that was associated with increased numbers of osteoclasts. These authors also demonstrated increased in vitro osteoclast differentiation of FcγRIII^−/−^ bone marrow cells, together suggesting an inhibitory role for FcγRIII in osteoclastogenesis [48]. They stated that the mechanistic basis for this surprising finding could be the sequestration of the γ-chain by FcγRIII. Therefore, in the absence of FcγRIII, more γ-chain is available for other proteins, such as osteoclast-associated immunoglobulin-like receptor (OSCAR) and paired immunoglobulin-like receptor A (PIR-A), which both act as costimulatory factors during osteoclastogenesis via their association with the γ-chain. In addition, the same research group showed that FcγRIIb^−/−^ cells, which lack the ITIM domain that inhibits pro-osteoclastogenic ITAM signaling, showed increased osteoclastogenic potential. Therefore, in the present study, where we show that FcγRI,II,III,IV^−/−^ cells have normal in vitro osteoclastogenic potential, we cannot rule out a compensatory effect on osteoclast differentiation via γ-chain-dependent costimulatory pathways, such as via OSCAR or PIR-A signaling, or via triggering receptor expressed on myeloid cells 2 (TREM2), which depends on the ITAM-domain containing DAP12 protein for its signaling. In line with this possibility, it has been shown that DAP12 is primarily responsible for in vitro M-CSF- and RANKL-induced osteoclastogenesis because γ-chain^−/−^ cells show normal differentiation. However, in the absence of DAP12, the γ-chain can compensate for its absence, acting via α_v_β_3_ integrin [49].

Finally, underlining the normal osteoclast differentiation that we observed in vitro, nonarthritic FcγRI,II,III,IV^−/−^ mice had no basal bone phenotype in their knee joints, measured as the amount of subchondral calcified tissue in the femur and tibia, and we observed comparable numbers of TRAP^+^ osteoclasts along the bone surface after AIA induction. Together, these findings imply that the absence of all FcγRs does not impair the ability of cells to differentiate into mature osteoclasts, suggesting that a difference in osteoclast activity, rather than differences in osteoclast numbers, must underlie the decreased erosion in FcγRI,II,III,IV^−/−^ mice in the studied AIA model.

It has been shown that inflammation plays a critical role in the activation of osteoclasts in vivo by the production of a plethora of factors that in this way lead to bone erosion. In this study, we found that inflammation (both infiltrate and exudate) was decreased in FcγRI,II,III,IV^−/−^ mice compared with WT controls at day 7, but not at day 21, after AIA induction. This suggests that FcγRs are probably most involved in the early inflammatory response of the AIA model, which still resulted in significantly decreased bone erosion in the FcγRI,II,III,IV^−/−^ mice at day 21 after induction.

It has been shown that the four FcγRs in mice are differentially expressed on immune cells and that the individual FcγRs are known to bind the various IgG subclasses with different affinities [10]. The activity of IgG1 is dependent mainly on FcγRIII, and IgG2a binds with high affinity to FcγRI and with low affinity to FcγRIII and IV, whereas IgG2b bind with the highest affinity to FcγRIV. This implies that the absence of one or more FcγRs and the cellular composition might facilitate the binding of IgGs to other FcγRs more than normally would occur, possibly leading to abnormal intracellular signaling. Previous researchers have investigated the role of FcγRs in AIA using mice deficient in one or more FcγRs. Induction of AIA in FcγRIIb^−/−^ mice resulted in increased inflammation, most probably because of the absence of both FcγRIIb-mediated IgG clearance and a negative feedback loop on IgG production by B cells [28, 29]. In contrast, the inflammation in FcγRI^−/−^ or FcγRIII^−/−^ mice was not affected [28], suggesting that either these receptors can compensate for each other’s absence or that FcγRIV is the dominant FcγR in both cases. However, the combined absence of FcγRI and FcγRIII [24] led to reduced inflammation. Interestingly, when combined with the absence of the IgG-clearing FcγRIIb, resulting in FcγRI,II,III^−/−^ mice, stronger accumulation of ICs is present, which results in increased inflammation, probably via binding of ICs to FcγRIV [29]. The important function of FcγRIV in this situation is substantiated by the finding that the inflammation is reduced when, in addition to FcγRI, FcγRII, and FcγRIII, FcγRIV is also absent. Although our results indicate an important role for FcγRIV in the pathology of AIA, definitive proof should come from AIA induction in an FcγRIV single-knockout. Consistent with our findings in the AIA model, the fact that 35–40% of FcγRI,II,III^−/−^ mice developed CIA, whereas γ-chain^−/−^/FcγRIIb^−/−^mice that also lack functional FcγRIV were protected from disease development, further supports the role of FcγRIV in this arthritis model [44]. Moreover, a clear confirmation for the crucial involvement of FcγRIV in K/B×N comes from a study by Seeling and colleagues in which FcγRIV^−/−^ animals showed significantly decreased inflammation and bone erosion [41]. Finally, arthritis development can be prevented using a blocking antibody against FcγRIV in mice lacking all FcγRs except for FcγRIV [50].

Together, these studies and the data reported in the present article show that an important role can likely be attributed to FcγRIV in experimental RA models. Interestingly, a polymorphism in FcγRIIIA, which is the human ortholog of the murine FcγRIV, has been associated with increased susceptibility to RA development. This polymorphism, in which a phenylalanine is substituted for a valine at amino acid position 158 (158 V/F), located at the immunoglobulin-binding domain, results in an increased affinity of the receptor for IgG1 and IgG3 antibodies [51–53]. Although these associations could not be reproduced in all populations, these data support a possible important role for the human ortholog of murine FcγRIV in RA development and further support the importance of additional studies to investigate the role of FcγRs in RA [54].

In our present study, we describe a clear increase in the numbers of neutrophils in both the infiltrate and exudate in FcγRI,II,III^−/−^ mice, whereas decreased neutrophil numbers were observed in FcγRI,II,III,IV^−/−^ mice. This suggests that particularly FcγRIV is responsible for the presence of neutrophils to the joint. The importance of neutrophils in RA development has been shown in the K/B×N experimental RA model [31]. Moreover, in agreement with this role for neutrophils in experimental RA models, high numbers of neutrophils are present in the joints of patients with active RA [32, 33]. We show a strong and significant correlation between the number of neutrophils present in the joint and the amount of bone erosion. Because in the AIA model ICs are produced locally in the joint, we propose that ICs can stimulate synovial macrophages via FcγRIV to release chemotactic factors such as complement components and keratinocyte-derived chemochine, thereby inducing the recruitment of neutrophils into the joint. Neutrophils are strong producers of the alarmin S100A8/A9, which has been shown to directly induce osteoclast activity in vitro via TLR4 signaling [39]. This suggests a mechanism through which infiltrated neutrophils can regulate bone erosion in RA. Indeed, we observed lower expression levels of S100A8 coinciding with lower neutrophil numbers in the joints of FcγRI,II,III,IV^−/−^ mice than in WT mice. Moreover, we observed that the production of S100A8 by neutrophils strongly and significantly correlated with the severity of bone erosion. In agreement with these findings, our laboratory previously showed that FcγRI,II,III^−/−^ mice, which have increased numbers of neutrophils in their joints after AIA induction, showed increased S100A8 expression. S100A8/A9 has been shown to be strongly upregulated in the synovial fluid of patients with RA [37, 38], and its levels are linked to joint inflammation and damage [55–58]. Together, these data support the idea that FcγRIV mediates bone erosion in AIA by modulating the influx of S100A8/A9-producing neutrophils into the arthritic joint, although an additional direct effect of ICs on osteoclasts cannot be completely ruled out.

## Conclusions

Our present study adds important new data to the existing body of knowledge concerning the involvement of FcγRs in inducing bone erosion and particularly highlights, for the first time to our knowledge, the role of FcγRIV in neutrophil-mediated bone erosion during AIA.

## Additional files

Additional file 1: Graphical representation of bone erosion scoring method and quantification of noncartilage collagenous tissue and proteoglycan (PG) depletion. **a** Graphical representation of the 13 locations along the patella, femur, tibia, and cruciate ligament where bone erosion was scored. **b** Quantification of the noncartilage collagenous tissue (blue staining in Safranin O/Fast Green staining) in femur and tibia showed no differences between naive FcγRI,II,III,IV^−/−^ and wild-type (WT) mice. *ns* Not significant. **c** Quantification of PG depletion showed a significant decrease at the tibiofemoral area in the joints of FcγRI,II,III,IV^−/−^ mice as compared with their WT controls (*n* = 10 and 14 joints per group, respectively) at both 7 and 21 days after AIA induction. Scatterplots are shown, with horizontal and vertical lines showing mean ± SEM values. *ns* Not significant. * *P* < 0.05, ** *P* < 0.01 versus WT controls. (PDF 437 kb)

Additional file 2: Gating strategy for flow cytometric analysis. Gating strategy for flow cytometric analysis used to identify CD11b^pos^Ly6C^high^ and CD11b^low/neg^Ly6C^high^ osteoclast precursor populations. First, single cells were selected. For identification of CD11b^pos^Ly6C^high^ monocytes, cells negative for CD90.2, CD45R/B220, CD49b, NK1.1, and Ly6G and positive for CD11b were selected (gate A). Subsequently, cells were back-gated for side scatter and forward scatter to exclude cells with high granulosity (gate B), and finally Ly6C^high^ cells were selected (gate C). For identification of CD11b^low/neg^Ly6C^high^, after exclusion of CD90.2-, CD45R/B220-, CD49b-, NK1.1-, Ly6G-positive cells (gate D), cells were gated for their expression of CD11b and Ly6C (CD11B^low/neg^Ly6C^high^) (gate E). (PDF 299 kb)

Additional file 3: NIMPR14- and F4/80-positive cells in the infiltrate and in the exudate in the joints of FcγRI,II,III,IV^−/−^ mice and their WT controls. Representative photomicrographs of (**a**) NIMPR14 and (**b**) F4/80 staining showing neutrophils and macrophages in the infiltrate and exudate of the knee joints of FcγRI,II,III,IV^−/−^ mice and their WT controls at day 7 after induction of antigen-induced arthritis. Original magnification ×400 for infiltrate and ×200 and ×400 for exudate. (PDF 422 kb)

Additional file 4: NIMPR14- and F4/80-positive cells in the infiltrate and exudate in the joints of FcγRI,II,III^−/−^ mice and their WT controls. Representative photomicrographs of (**a**) NIMPR14 and (**b**) F4/80 staining showing neutrophils and macrophages in the infiltrate and exudate of the knee joints of FcγRI,II,III^−/−^ mice and their WT controls at day 7 after induction of antigen-induced arthritis. Original magnification ×400 for infiltrate and ×200 and ×400 for exudate. (PDF 401 kb)

## Abbreviations

AIA: Antigen-induced arthritis
CFA: Complete Freund’s adjuvant
CIA: Collagen-induced arthritis
DAP-12: DNAX activation protein of 12 kDa
FcγR: Fcγ receptor
*Gapdh*: Glyceraldehyde 3-phosphate dehydrogenase
IC: Immune complex
IgG: Immunoglobulin G
ITAM: Activation motif immunoreceptor tyrosine-based activation motif
ITIM: Immunoreceptor tyrosine-based inhibitory motif
mBSA: Methylated bovine serum albumin
M-CSF: Macrophage colony-stimulating factor
mRNA: Messenger RNA
ns: Not significant
OSCAR: Osteoclast-associated immunoglobulin-like receptor
PE: Phycoerythrin
PG: Proteoglycan
PIR-A: Paired immunoglobulin-like receptor A
RA: Rheumatoid arthritis
RANKL: Receptor activator of nuclear factor-κB ligand
RF: Rheumatoid factor
TRAP: Tartrate-resistant acid phosphatase
TREM2: Triggering receptor expressed on myeloid cells 2
WT: Wild type
