# Supplementary material for: Fcγ receptor-mediated influx of S100A8/A9-producing neutrophils as inducer of bone erosion during antigen-induced arthritis
Source: Arthritis Res Ther. 2018 May 2;20:80. doi: 10.1186/s13075-018-1584-1 (PMC5932875; doi:10.1186/s13075-018-1584-1)
Supplement: Supplementary file 1 — Graphical representation of bone erosion scoring method and quantification of noncartilage collagenous tissue and proteoglycan (PG) depletion. a Graphical representation of the 13 locations along the patella, femur, tibia, and cruciate ligament where bone erosion was scored. b Quantification of the noncartilage collagenous tissue (blue staining in Safranin O/Fast Green staining) in femur and tibia showed no differences between naive FcγRI,II,III,IV−/− and wild-type (WT) mice. ns Not significant. c Quantification of PG depletion showed a significant decrease at the tibiofemoral area in the joints of FcγRI,II,III,IV−/− mice as compared with their WT controls (n = 10 and 14 joints per group, respectively) at both 7 and 21 days after AIA induction. Scatterplots are shown, with horizontal and vertical lines showing mean ± SEM values. ns Not significant. * P < 0.05, ** P < 0.01 versus WT controls. (PDF 437 kb) [file 13075_2018_1584_MOESM1_ESM.pdf]

Additional file 1

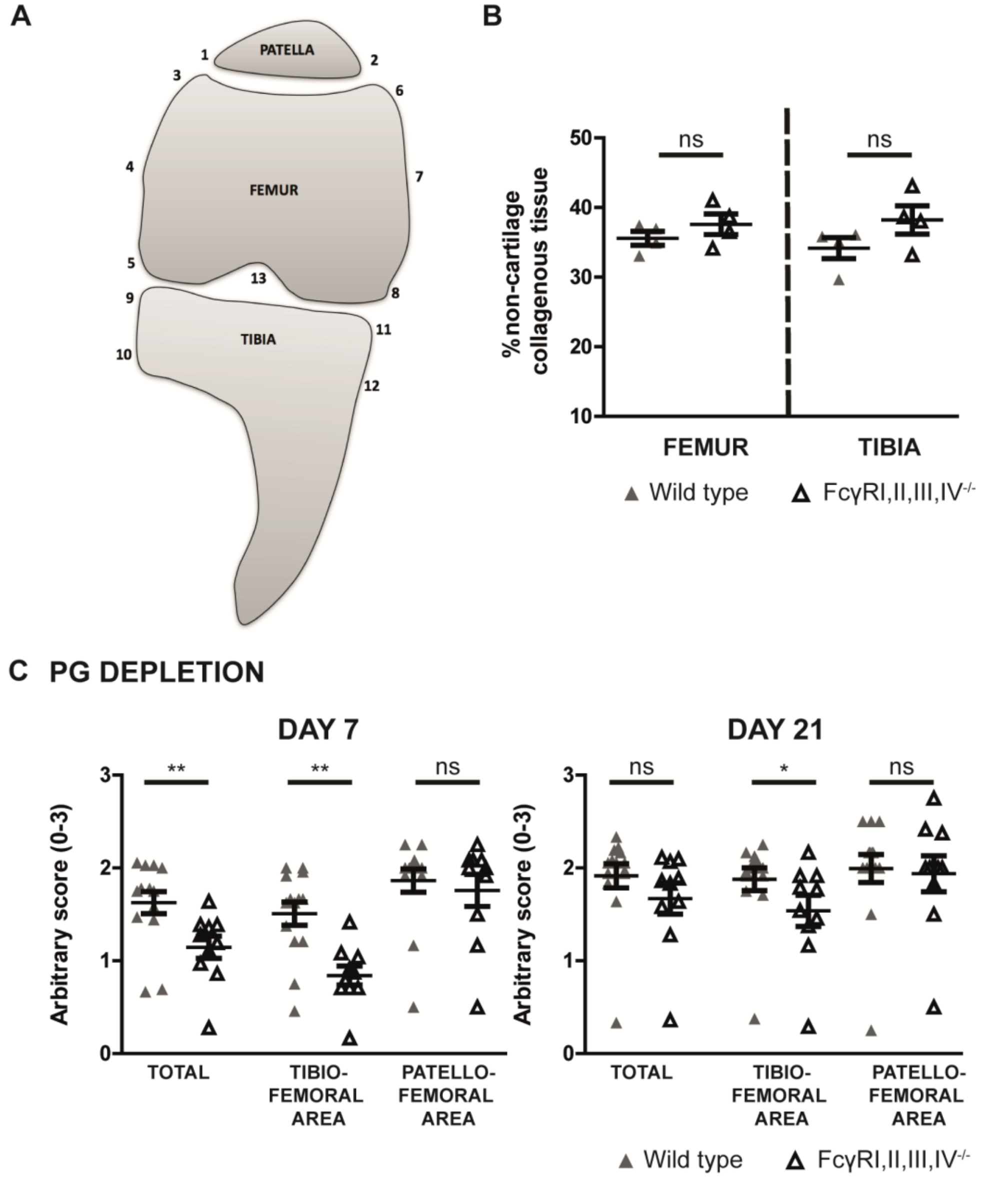

Graphical representation of bone erosion scoring method, quantification of non-cartilage collagenous tissue and proteoglycan depletion.

A) Graphical representation of the 13 locations along the patella, femur, tibia and cruciate ligament where bone erosion was scored. **B)** Quantification of the non-cartilage collagenous tissue (blue staining in Safranin O/Fast Green staining) in femur and tibia showed no differences between naive FcγRI,II,III,IV$^{-/-}$ and wild type mice. ns=not significant. **C)** Quantification of PG depletion showed a significant decrease at the tibio-femoral area in the joints of FcγRI,II,III,IV$^{-/-}$ mice as compared to their WT controls (n=10 and 14 joints per group respectively) at both 7 and 21 days after AIA induction. Scatter plots are shown, with horizontal and vertical lines showing mean +/- SEM values. ns=not significant; * = $P < 0.05$ ** = $P < 0.01$; versus WT controls.
