# Supplementary material for: Fcγ receptor-mediated influx of S100A8/A9-producing neutrophils as inducer of bone erosion during antigen-induced arthritis
Source: Arthritis Res Ther. 2018 May 2;20:80. doi: 10.1186/s13075-018-1584-1 (PMC5932875; doi:10.1186/s13075-018-1584-1)
Supplement: Supplementary file 2 — Gating strategy for flow cytometric analysis. Gating strategy for flow cytometric analysis used to identify CD11bposLy6Chigh and CD11blow/negLy6Chigh osteoclast precursor populations. First, single cells were selected. For identification of CD11bposLy6Chigh monocytes, cells negative for CD90.2, CD45R/B220, CD49b, NK1.1, and Ly6G and positive for CD11b were selected (gate A). Subsequently, cells were back-gated for side scatter and forward scatter to exclude cells with high granulosity (gate B), and finally Ly6Chigh cells were selected (gate C). For identification of CD11blow/negLy6Chigh, after exclusion of CD90.2-, CD45R/B220-, CD49b-, NK1.1-, Ly6G-positive cells (gate D), cells were gated for their expression of CD11b and Ly6C (CD11Blow/negLy6Chigh) (gate E). (PDF 299 kb) [file 13075_2018_1584_MOESM2_ESM.pdf]

Additional file 2

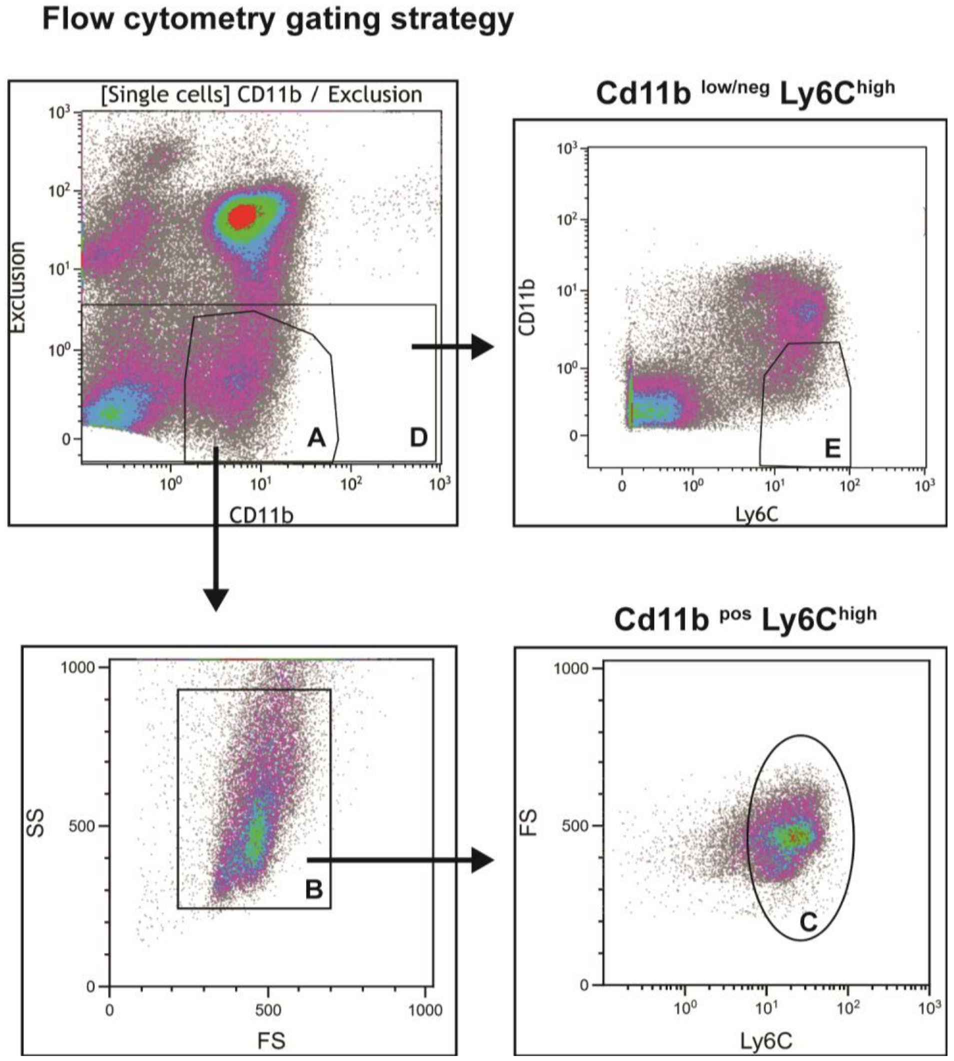

Gating strategy for flow cytometry analysis

Gating strategy for flow cytometry analysis used to identify CD11b^{pos} Ly6C^{high} and CD11b$^{low/neg}$ Ly6C^{high} osteoclast precursor populations. First, single cells were selected. For identification of CD11b^{pos} Ly6C^{high} monocytes, cells negative for CD90.2, CD45R/B220, CD49b, NK1.1, Ly6G and positive for CD11b were selected (gate A). Subsequently, cells were back gated for side scatter and FS to exclude cells with high granulosity (gate B) and finally Ly6C^{high} cells were selected (gate C). For identification of CD11b$^{low/neg}$ Ly6C^{high} , after exclusion of CD90.2, CD45R/B220, CD49b, NK1.1, Ly6G positive cells (gate D), cells were gated for their expression of CD11b and Ly6C (CD11B$^{low/neg}$ Ly6C^{high}) (gate E).
