# Supplementary material for: Fcγ receptor-mediated influx of S100A8/A9-producing neutrophils as inducer of bone erosion during antigen-induced arthritis
Source: Arthritis Res Ther. 2018 May 2;20:80. doi: 10.1186/s13075-018-1584-1 (PMC5932875; doi:10.1186/s13075-018-1584-1)
Supplement: Supplementary file 3 — NIMPR14- and F4/80-positive cells in the infiltrate and in the exudate in the joints of FcγRI,II,III,IV−/− mice and their WT controls. Representative photomicrographs of (a) NIMPR14 and (b) F4/80 staining showing neutrophils and macrophages in the infiltrate and exudate of the knee joints of FcγRI,II,III,IV−/− mice and their WT controls at day 7 after induction of antigen-induced arthritis. Original magnification ×400 for infiltrate and ×200 and ×400 for exudate. (PDF 422 kb) [file 13075_2018_1584_MOESM3_ESM.pdf]

Additional file 3

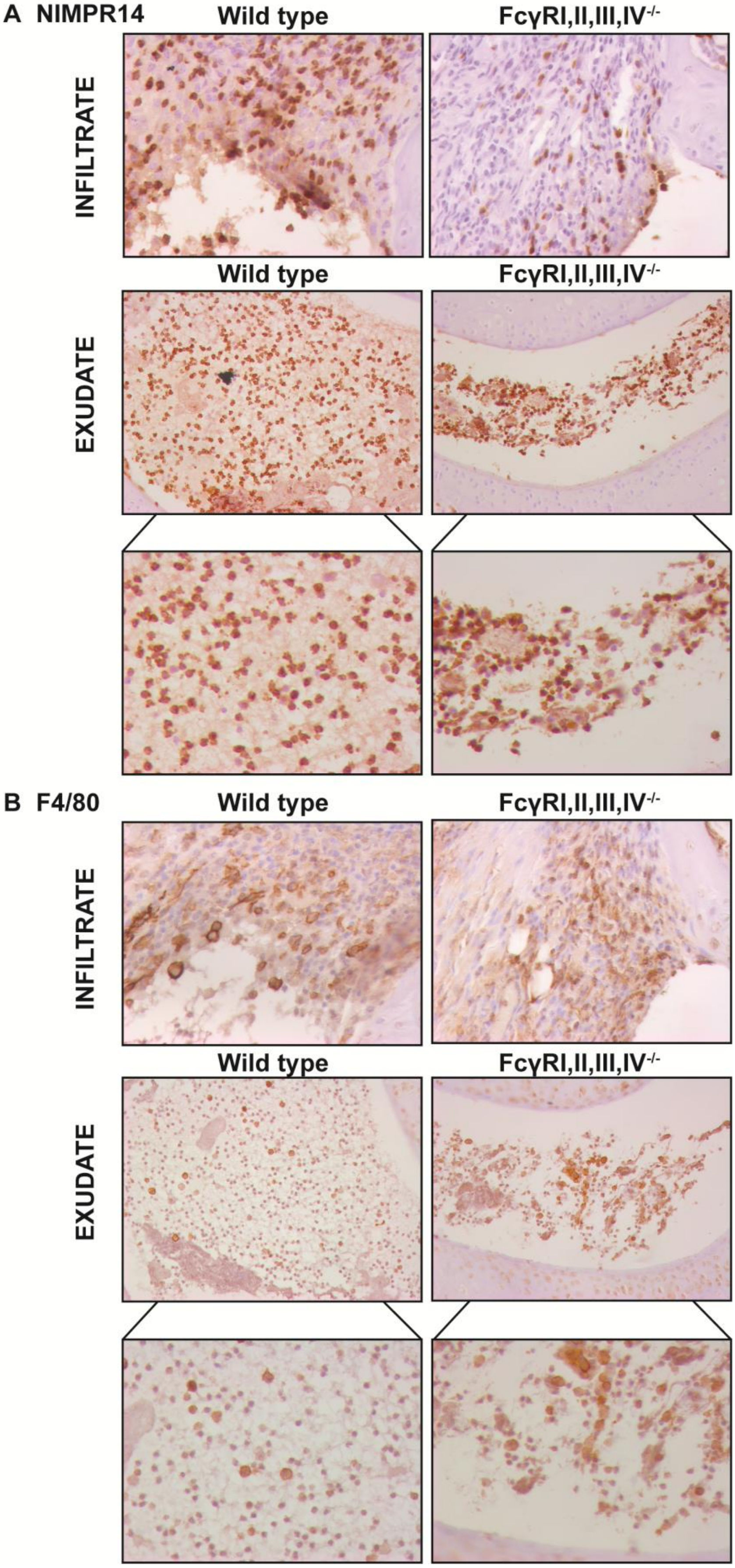

NIMPR14 and F4/80 positive cells in the infiltrate and in the exudate in the joints of FcγRI,II,III,IV$^{-/-}$ mice and their WT controls.

A) Representative photomicrographs of NIMPR14 and **B)** F4/80 stainings showing neutrophils and macrophages in the infiltrate and in the exudate of the knee joints of FcγRI,II,III,IV$^{-/-}$ mice and their WT controls at day 7 after induction of antigen-induced arthritis. Original magnification 400X for infiltrate and 200x and 400x for exudate.
